# Identification of Insertion Deletion Mutations from Deep Targeted Resequencing

**DOI:** 10.4172/2153-0602.1000132

**Published:** 2013-07-02

**Authors:** Georges Natsoulis, Nancy Zhang, Katrina Welch, John Bell, Hanlee P Ji

**Affiliations:** 1 Division of Oncology, Department of Medicine, Stanford University School of Medicine, Stanford, CA, 94305, USA; 2 Department of Statistics, University of Pennsylvania, Philadelphia, PA, 19104, USA; 3 Stanford Genome Technology Center, Stanford University, Palo Alto, CA, 94306, USA

## Abstract

Taking advantage of the deep targeted sequencing capabilities of next generation sequencers, we have developed a novel two step insertion deletion (indel) detection algorithm (IDA) that can determine indels from single read sequences with high computational efficiency and sensitivity when indels are fractionally less compared to wild type reference sequence. First, it identifies candidate indel positions utilizing specific sequence alignment artifacts produced by rapid alignment programs. Second, it confirms the location of the candidate indel by using the Smith-Waterman (SW) algorithm on a restricted subset of Sequence reads. We demonstrate that IDA is applicable to indels of varying sizes from deep targeted sequencing data at low fractions where the indel is diluted by wild type sequence. Our algorithm is useful in detecting indel variants present at variable allelic frequencies such as may occur in heterozygotes and mixed normal-tumor tissue.

## Introduction

Recent advances in DNA sequencing technologies are revolutionizing genetics and genomics [1]. Improvements in next generation DNA sequencers have dramatically increased the output of sequence and, as a result, enabled large scale resequencing studies to determine, en masse, polymorphisms, rare variants and somatic mutations. As a result, many groups are pursuing the analysis of specific gene sets from the genome using deep targeted resequencing. This involves selectively enriching specific gene targets and sequencing these genes with very high fold coverage in the hundreds if not higher. An important category of genetic variation is insertions/deletions (indels), which represent a significant proportion of the genetic diversity of the human genome. Likewise, indels account for approximately 7% of the coding region mutations in some cancers, as the recent study of all of the known coding regions of colorectal and breast cancers demonstrated [2]. The detection of indels in specific genes is of particular interest given their effects in producing stop codons, out-of-frame coding changes or splice site alterations, all of which have dramatic effects on normal protein function [3].

For normal germline genomes, heterozygote indels are typically represented among approximately 50% of the sequence reads from a given gene target. However, there are many examples where samples are mixed and a particular indel may be represented in lower fraction compared to the wild type sequence. This commonly occurs in cancer where normal tissue may dilute cancer genomic regions and viral genomes where mutations may be specific to a specific sub-strain. A number of next generation sequencing alignment tools for single read alignment have been specially developed for next generation sequencers [1,3]. The majority of these alignment tools can detect mismatches secondary to single nucleotide polymorphisms (SNPs) and small indels [3]. However, these rapid alignment tools may lack sensitivity to detect indels of varying frequencies from heterozygotes and heterogeneous mixtures of genotypes as occurs in tumor mixed with normal tissue and require additional post-alignment processing for indel discovery [3].

Given the need for improving the detection of indels from deep targeted resequencing data of gene subsets, we developed a two-step indel detection algorithm, hereafter referred to as IDA, which is capable of using single read alignment results and detecting indels at lower fractions than is what typically seen in normal germline genome sequencing. Leveraging the increased output of next generation sequencers, IDA relies on very high fold coverage in the hundreds of specific regions and is particularly useful for targeted resequencing data sets of specific genes. First, IDA identifies a set of candidate indel positions by taking advantage of sequencing depth aberrations and a characteristic pattern of mismatches produced by fast sequence alignment algorithms. Second, IDA uses the Smith-Waterman algorithm (SW) to confirm the presence of indels in the vicinity of each candidate position. Overall, the advantages of our algorithm include: 1) detection of indels of variable sizes from sequence data, 2) detection of indels that are carried by only a fraction of the cells, as might occur in mixed tumor-normal tissues with somatic mutations, 3) straightforward integration at the end of rapid alignment processes. IDA is particularly useful in targeted resequencing applications in which specific regions of the genome as with PCR amplicons are being sequenced with very high fold coverage to identify mutations at higher sensitivity.

## Methods

### Genomic DNA samples

The PhiX174 control sample provided with the Illumina sample preparation kit. Genomic DNA was extracted from three colorectal cancer cell lines with known indels locationed in exons 7, 8 and 9 (HCA7, SW1417 and COLO741). A fourth cell line without indels in these specific *TP53* exons was also sequenced (COLO201). The colorectal cancer cell lines were grown with 10% FBS (Autogen Bioclear, Wiltshire, U.K.) and 6 mM L-glutamine. All cultures were mycoplasma free and maintained in a humidified atmosphere with controlled CO2 content as indicated. Genomic DNA was extracted from the colorectal cancer cell lines by using the DNeasy Tissue Kit (Qiagen, Crawley, U.K.) following the manufacturer's protocols. Genomic DNA was isolated from peripheral leukocytes using the Gentra genomic DNA preparation kit (Minneapolis, MN).

### PCR amplification of TP53 from colorectal cancer cell lines

Two set of primers were designed to amplify exons 7, 8 and 9 of *TP53* (Supplemental Table 2). One amplicon covered exons 7 and while the second amplicon covered exons 8 and 9. The standard PCR was in a volume of 50 μl containing 50 ng template DNA, 10 pmol of each primer, 0.25 mM each dNTP, 2.5 mM MgCl_2_, 5 units of AmpliTaq Gold (Applied Biosystems), 1×Taq Gold buffer (Applied Biosystems), and the appropriate volume of H2O. The following PCR program was used to amplify the exons: 1 cycle of denaturation (12 min at 95°C), 15 cycles of denaturation (95°C for 30 s), annealing temperature (T m; +7.5°C for 30 s, with −0.5°C per cycle), and extension (72°C for 30s), followed by 20 cycles of denaturation (95°C for 30s), annealing (T m, 30s), and extension (72°C for 30s), and a final extension cycle of 72°C for 10 min.

### DNA sequencing

The samples were processed according to the manufacturer's specifications. This included the fragmentation step prior to preparation of the sequencing libraries. The sequencing flow-cell was processed according to the Illumina Cluster Station protocol and the sequencing protocol. Images were collected and after the run, image analysis, base calling and error estimation were performed using Illumina sequencing software (vs. 0.2.2.6).

### Data sets

For the initial development of IDA, we used sequence data sets from two sources. The first was generated from the previously described sequencing of the phix174 genome. We chose a subset of sequence reads covering 50 separate positions, each spaced 100 bp apart and ranging from positions 100 to 5,000 in the phix174 genome sequence (GI:9626372). For each subset of sequence reads covering the 50 positions, 25%, 50% or 100% of the sequence reads had a 1, 2 or 3 bp insertion randomly introduced. We refer to these introduced indels as “spiking” and the percentage of indel-containing reads as the spike fraction. The same insertion was used for each member of a given subset aligning with a specific position in the phiX174 genome. We used a similar approach to generate 1, 2 or 3 bp deletions. For the second data set, we simulated a random 10.1 Kbp sequence and created 20,000 Sequence reads randomly positioned from this simulated reference sequence. To simulate sequencing errors, 1 random mismatch was introduced in each fragment. This corresponds to a ∼3% error rate and is significantly higher than what we typically observe in real sequencing data. The distribution of the introduced mismatches within individual sequences is intended to mirror what we have observed in real sequencing data sets. Finally, for this simulated data set, we introduced indels at 100 coordinates spaced 100 bp apart from position 100 to 10,000. Only results for the 50% spike ratio are reported. For final confirmation of the utility of IDA on a human genomic sample, we analyzed sequence reads from amplicon sequencing of the *TP53* gene as derived from colon cancer cell lines.

### Definitions, notations and functions

To simplify our description, we will assume that the read data comes from the forward strand (3′-5′ direction) only. There is no loss of generality in making this assumption since sequence data which comes from the reverse strand can be treated as ata coming from the forward strand by using its reverse complement.

*X* will be used to refer to the reference (long) sequence against which we are aligning our sequence data. The bases in *X* take their values from a 4-letter alphabet A={*A*, *C*, *G*, *T*}. The base at the *i*^th^ position in the reference will be refered to as *X_i_*. For *j* ≥ *i*, $X_{i}^{j}$ will be used to refer to the consecutive bp of *X* that start at position i and end at position *j*. Thus, for example, if our reference sequence is ‘AAGTC’, then *X*_3_ =‘*G*’ while *X*_2_^4^ =‘*AGT*’.

We will use *y* to represent the individual read. In this analysis, we found that improved results can be obtained if the 8 bp on either side of the sequence are ignored in computing the depth function, and only the middle portion is taken. Thus, we define *ỹ* to be the middle portion of the read, and *y* to be the original read containing the end positions. Here, it will be convenient to give an arbitrary order to the sequenced reads and let *y_i_* denote the *i*^th^ read obtained by the sequencer. Note that, in contrast to the convention for *X*, *y_i_ ỹ _i_*) here refers to a sequence of bases rather than just 1 bp. Let S represent the set of reads obtained by the sequencer and let *N* represent the total number of reads obtained in one sequencing run/analysis that have been aligned successfully to *X* by a rapid alignment process. Hence, *S*=*{y_1_*, *y_2_*, …. *y_N_}*. The notation *y_i_*, *k (ỹ*_*i*, *k*_*)* refers to base *k* from (truncated) sequenced read i. Here, the position index *k* is taken with respect to the reference sequence *X* and we allow *y*_*i*,*k*_ to take on values in a 5-letter alphabet *B*=*{A*, *C*,*G*, *T*,*φ}* where *y_i_*, _*k*=_*φ* if the sequence *y_i_* does not align to *X* at the *K^t^*^h^ position. Hence for example, if X=‘AGGTTC’ and ‘GTT’ is aligned to X at position 3, then we can think of the sequence representation for ‘GTT’ as *y_1_*=‘*φφ GTT*’. The *i^t^*^h^ largest element of a vector: For x ∈ **R**^n^, we lwt x_[i]_ denote the *i^th^* largest component of *x* ; i.e. *x_[1]_* ≥ *x_[2]_* ≥ *K x_[i]_* ≥ *x_[i +1]_ Kx_[n]_*. As per standard convention, if H is a set, then | H | denotes the cardinality of the set. We further define several processes that run along the sequence *X*: We define the sequencing depth function as *d(k)*=| *{ŷ*_*i*, *k*_ *:1* ≤ *i* ≤ *N}* |. This function counts the number of sequences that align to the reference sequence at the *k* ^th^ position and is a representation of bp fold-coverage at any given nucleotide in the reference.

We define the matching function as follows: For *z* ∈ B, define *m_z_ (k)* =| *{y*_*i*, *k*_ *:1* ≤ *i* ≤ *N*, *y_i_*, _*k*_=*z}* |. This function counts the number of sequences that match the letter z at the k th position. Hence, the number of sequences that matches X_k_ is given by *m_x_k__ (k)*.

We define the mismatch vector as follows: at position *k*: Let *C*=*A* \ *X_k_* ; that is, the set A with the element corresponding to *X_k_* removed. C is therefore a 3 element set. Let *c*_1_, *c*_2_ and *c*_3_ denote the elements of the set. We define the mismatch vector at position *k* as *mv*(*k*) = [*m*_*c*_2__(*k*),*m*_*c*_2__(*k*),*m*_*c*_3__(*k*)]. Let *m*_[1]_ (*k*) be the frequency of the mismatched letter with greatest frequency at position *k*.

### IDA description

The indel detector algorithm or IDA consists of two steps, with the first step having a novel feature which greatly speeds up indel detection relative to using standard aligment algorithms such as Smith-Waterman alone. IDA was developed with Matlab (Mathworks).

**Figure 1:**
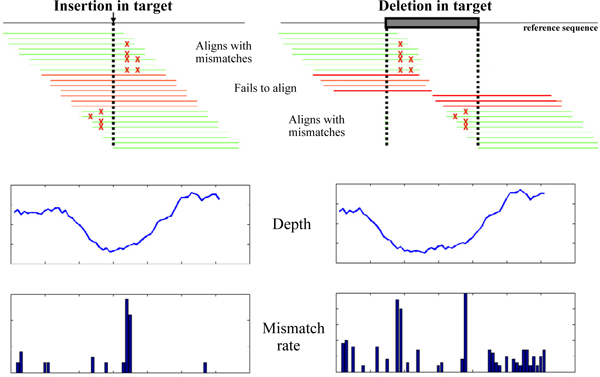
Principle of the indel detection algorithm Sequence reads are processed using a rapid alignment program (e.g.: MAQ, or ELAND) configured for identifying only mismatches. Green lines represent sequence reads which align through an edge effect. Red lines represent reads which fail to align. The position of the indel is indicated by an arrow (insertion) or a box (deletion). A portion of the sequences align through their edges and are reported erroneously as containing multiple mismatches. Most of the reads with an indel located in the center of the sequence fail to align. This causes the depth to drop in the region flanking the indel and the mismatch rate to rise at its boundaries, as shown in the example plots. These plots are example regions taken from the phix174 spike-in study.

In step 1 of our algorithm we rely on the sequence alignment data generated from ELAND or MAQ. These tools report sequence mismatches from the reference. For our algorithm, they are not explicitly configured for detecting indels. The key observation is that only a subset of sequence reads containing an indel can align to the reference. The sequence reads (containing an indel) that do align are comprised of those where the number of mismatches caused by the additional (insertion) or missing (deletion) bp(s) are within the limit allowed by the settings of the alignment program. Hence the mismatches are found at one end of the sequence (at the position of the indel and beyond as in Figure 1). The neighborhood of the indel exhibits two specific properties which we exploit in the first step of the algorithm.

(1) The sequencing depth *d*(*k*) (bp fold coverage) drops significantly around the indel location as compared to a sequenced region not containing an indel because only a limited subset of sequences align secondary to edge effects. The magnitude of the drop in depth increases with the proportion of reads containing the indel compared to the complete data set. The width of the drop depends on whether the indel is a deletion or insertion. Let L be the length of the sequenced reads. Consider the case of an insertion at point t. As shown in Figure 1, the depth is expected to drop linearly starting at t-L, reaching its lowest value at t, and rising linearly to its normal value at t+L. This follows simply from the definition of *d*(*k*) as the sum of the number of reads whose starting position falls within the window *[t*-*L*,*t*+*L]*, and thus an indel at *t* disallows alignments to the reference except those causing edge effects. The case of deletions is more complex. Let the deletion start at *t_1_* and end at *t_2_* in the reference genome. As shown in Figure 1, the depth drops linearly starting at *t_1_*-*L*, reaching its lowest value at *t_1_*, stays at this lowest value until *t_2_*, then rises linearly to its normal value at *t_2_*+*L*. Thus, unlike insertions, the width of the drop in depth depends on the width of the deletion.

(2) Of aligned sequence reads, the proportion with mismatches at their edges increases significantly around the indel location. For insertions, this increase occurs immediately to the left and right of the point of insertion in the reference genome. For deletions, this increase occurs immediately to the right of the start and immediately to the left of the end of the deleted segment. If the mismatch is caused by an indel, as opposed to a sequencing error, then the highest mismatch letter count *m*_[1]_ (*k*) should be much higher than the second highest mismatch letter count *m*_[2]_ (*k*).

To insure that reads can adequately cover regions greater than 50 bp, the majority of next generation sequencers typically require a random fragmentation of the target DNA template. A number of extrinsic factors such as sequencing protocol variation influence the fold-coverage of any given region of sequence and the appearance of mismatches. As a result, for any given sequencing run, the sequencing depth and mismatch vector fluctuate significantly across positions in X, regardless of indels. To compensate, we normalize the sequencing depth and mismatch vector to make them comparable across the regions being sequenced. We found that the local median smoothed values, *d* (*k*) and *m*_[1]_ (*k*), provide an adequate control. Thus we define the local median smoothed depth function as: *d* (*k*)=median{*d* (*j*) : | *j* − *k* |< *w_d_*} and the local median smoothed mismatch count as *m*_[1]_ (*k*)=median{*m*_[1]_ (*j*) : | *j* − *k* |< *w_m_*}. In defining the median smoothed depth *d* (*k*) and mismatch vector *m*_[1]_ (*k*), we rely on pre-specified window sizes *w_d_* and *w_m_* respectively. In Supplemental Materials Section 3, we show that *w_d_* and *w_m_* should be chosen to be larger than 2L, where L is the read length. We will also show empirically that our method is robust with respect to values of *w_d_* and *w_m_* greater than 2L.

From this, we now define the normalized depth to be *d̂*(*k*) = *d*(*k*)/*d̅*(*k*) and the normalized mismatch vector to be

$${\hat{m}}_{\left[1\right]}(k)=[m_{\left[1\right]}(k)/{\bar{m}}_{\left[1\right]}(k)>2m_{\left[2\right]}(k)]$$

where the factor *I*[*m*_[1]_ (*k*) >2*m*_[2]_ (*k*)] is an indicator function which checks that the highest mismatch base count is more than twice the second highest mismatch base count as previously described.

In step 1 of IDA, the indel detector function is derived as follows. As noted in the results, we provide evidence that *d̃* (*k*) decreases and *m̃*_[1]_ (*k*) increases near the position of indels. To make use of the information value of both these effects, a window-smoothed ratio function *f*(*k*), defined as follows, is used:

$$f(k)=\sum_{j=k-w_{f}}^{k+w_{f}}{\hat{m}}_{\left[1\right]}(j)/\hat{d}(k)$$

should increase near an indel location. *f(k)* is referred to as the “indel detector” function. The first step of the algorithm identifies candidate indels to be all locations *k* where *f*(*k*) >*f_THRESH_* for a predetermined threshold *f_THRESH_*.

In step 2 of IDA, SW is used to confirm candidate indel location. From the set of reads that did not align in the initial MAQ alignment, IDA first identifies those individuals reads that match the flanking regions of the candidate position. IDA does this for each candidate indel position generated from step 1. This subset S_0_ of reads is then aligned using SW against a short segment of the reference sequence comprising the candidate position. We use the candidate indel position *p* +/- 40 bp. A candidate position is deemed confirmed if SW aligns more than M (we have used M=4) out of the S_0_ reads with the reference segment while introducing a shared indel less than 5 bp from position *p* and with fewer than two mismatches. By shared indel we refer to SW introducing an indel in the exact same position in more than M instances. That position, which may differ from the candidate position, defines the confirmed indel position.

## IDA Pseudo-code

**First step** Inputs: *X*, window sizes *w_d_*, *w_m_*, and *w_f_*, threshold *f_THRESH_*, sequence data S Outputs: SUSPECT, the set of candidate indel locations For *k*=*w _f_* + 1, …,*T* − *w_f_*, where *T* is the length of the reference sequence, compute the function *f*(*k*) as defined above. Let *SUSPECT* ←{*k* : *f* (*k*) > *f_THRESH_*}**Second step** Inputs: *X*, *SUSPECT*, *S*_0_, *M* Outputs: INDEL, the set of indel locations INDEL ← EMPTY **For** all *k* ∈ *SUSPECT*
**do**
*testseq* ← 
$X_{k-40}^{k+40}$ **For** all *y_i_* ∈ *S*
**do** Align *y_i_* and testseq using Smith-Waterman (SW) algorithm. **end for** **if** more than *M y_i_* align to *testseq* with less than 2 mismatches and with shared indel interval [*p*, *q*], **then** INDEL ← INDEL ∪ {[*p*, *q*]}  **end if end for**

## Results

### Algorithm description

IDA is applied to postaligned data. These tools report alignment positions and mismatches from the reference and are not explicitly configured for detecting indels. Our careful examination of alignment results in the vicinity of indels revealed two artifacts that are the basis of step 1 of the IDA algorithm (Figure 1). First, the fold coverage, otherwise referred to as sequencing depth, *d* (*k*), drops around the 9 indel location. Most indel-containing reads fail to align when the indel is located close to the center of the read. Second, a subset of indel-containing reads align to the reference through “edge effects” where the indel is located at one end of the reads. The size of this subset depends on the tolerance limit for mismatches of the alignment program. As shown in Figure 1, as a result of this edge effect, the overall sequencing depth does not drop to zero even in the presence of an indel. We define an indel detector function, *f(k)*, which is a ratio of the normalized depth to the normalized mismatch functions (for more details, see Methods). In the vicinity of indels, *f(k*) increases, thus identifying candidate indel positions. For the indel detector function, *f(k)*, we calculate the sequencing depth using only the middle portion of each read, trimming eight bps at either end of the sequence read. As a result, this specialized variation on the depth function does drop to zero in the presence of an indel when that indel is present in 100% of the reads. We found that this substantially improves the sensitivity of the algorithm.

In step 2 of IDA, we confirm the presence of indels in the vicinity of the candidate positions that were identified in step 1 using the indel detector function. The confirmation utilizes a gapped alignment procedure such as the SW algorithm. The second step of IDA focuses on the subset of reads that completely failed to align. SW is used to align these readson the short segments of reference sequence surrounding the candidate indel positions. A candidate indel is confirmed if at least *M* of the reads that align to its neighborhood shares a common gap. Step 2 of IDA uses only a limited number of reads from the set of sequenced reads and a small segment of reference sequence encompassing the putative indel position. Because the requirements of a SW-based computation are proportional to these two aforementioned factors, IDA achieves a commensurate reduction in computation time. More details of the IDA algorithm, including computation of the indel detector function and parameter settings are reported in the Methods.

### Receiver operating curve (ROC) analysis of the algorithm

We evaluated the performance of IDA on an experimental data set generated from an Illumina Genome Analyzer (Table 1) and on *in-silico* generated sequence reads (Supplemental Material Section 2). For the experimentally generated reads, we used a portion of the data produced in a single lane of sequencing of the phix174 genome (76,247 reads). This subset resulted in average sequencing fold coverage of 396 based on the 5,386 bp size of phiX174 genome. For our ROC analysis, a large number of conditions were tested repeatedly, justifying the use of a subset of the complete sequence generated from an entire run. However, we found that IDA runs on a complete data set from an Illumina Genome Analyzer sequencing lane (typically >10 million sequence reads) in approximately 50 seconds (Supplemental Material Section 1). We identified reads overlapping with 50 locations spaced at 100 bp intervals across the phix174 genome. For each location, an indel 1, 2 or 3 bp in length was randomly introduced among 100%, 50% or 25% of the reads covering these specific locations. This distribution models a diploid homozygous indel (100%), a diploid heterozygous indel (50%) or a somatic heterozygous indel (25%) present in a tumor where the tumor tissue contains a heterozygous somatic indel but tumor represents only 50% of the sample. We refer to the introduced percentage of indel-containing reads as the “spike fraction”. The entire sequence data set including the indel-containing reads were aligned against the phiX174 reference genome. The alignment results were parsed then submitted to the indel detection algorithm. As per the algorithm, a confirmed indel fulfilled the following criteria: 1) passing the threshold level generated by the first step, 2) confirmation by SW in the second step and 3) SW identification of a shared indel within 5 bp of the introduced indel position. In order to compare data sets containing different number of indels such as those listed in Table 1 and in Supplemental Table 1, in each case, up to eight candidate indel positions were submitted for the second step of the algorithm for each true indel position.

The receiver operating characteristic (ROC) analysis (Table 1) shows that indels within 1 to 3 bp in length are detected with high sensitivity. Even at an indel spike fraction of 25%, we observe sensitivities of 54% or better for indels 1, 2, or 3 bp in length. At 100% spike fraction, sensitivities were 100%. Specificities of approximately 100% are observed for all sizes and spike ratios. This high specificity is imparted by SW in the second step of the algorithm. Except for the computational cost, one could reach higher sensitivities even in the 25% spiked indel proportion simply by testing more candidates.

### Detecting variable length indels

**Figure 2:**
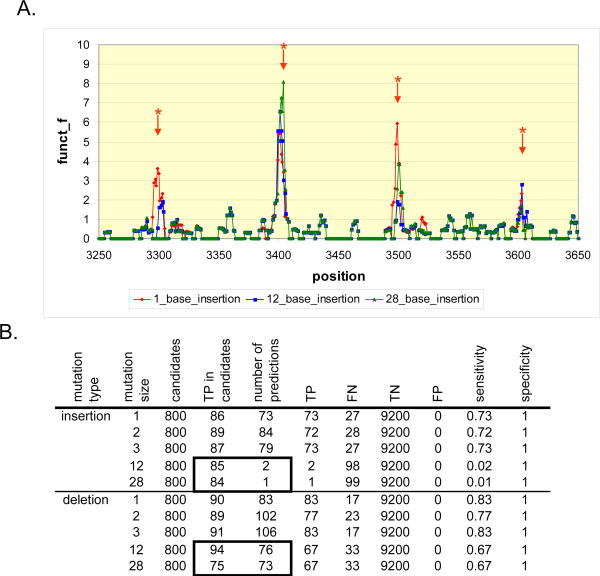
Analysis of results from step one of the indel algorithm. The first step of the algorithm can detect indels of any size Insertions of increasing size were introduced in varying percentages from reads derived from a 10.1 Kbp sequence (Supplemental Materials Section 2). Afterwards, the entire sequence data set was aligned with MAQ, and subsequently, IDA was applied for determining the location of these insertions. A. The X axis represents the position along the reference sequence in bp. The Y axis is the detector function *f*(*k*). It rises at the position of insertions (*), even when the insertion size is equal to the read length of 28 bp. B. The overall sensitivity of the algorithm drops for insertions greater or equal to 12 bp. This effect is due to the failure of SW to confirm candidate positions correctly identified by the first pass of the algorithm. This can be seen by inspecting the boxed area of the table. An equivalent number of true indels are detected for lengths 1 to 28 bp. However, for insertions, most of these candidates are not confirmed by the step 2 of IDA and very few predictions are made. True positives, false negatives, true negatives and false positives are annotated as TP, FN, TN and FP respectively.

We examined the sensitivity of detecting indels of variable length (Supplemental Table 1). Deletions varying from 1 to 28 bp and insertions from 1 to 3 bp in length are efficiently detected. Setting the extend gap parameter of the SW algorithm to zero is important to allow confirmation of deletions greater than 3 bp but does not apply to insertions greater than 3 bp (Supplemental Material Section 2). Large insertions are readily detected by the first step of the algorithm but SW is limited in confirming the exact location due to the short read lengths (Figure 2). One potential approach to improve the detection of large insertions is the use of a localized assembly for the second step instead of SW.

As noted in Figure 1 and in the Methods, for deletions the width of the drop in depth depends on the length of the deletion. Thus, for longer deletions larger window sizes *w_d_* and *w_m_* would yield higher sensitivity. We show that these values should be set to at least *2L* (where *L* is the length of an individual read), and that our method is reasonably robust to values significantly beyond *2L* (Supplemental Material Section 3).

### Setting the threshold for the first step of the algorithm

In the ROC analysis described above, we evaluated IDA's performance by testing a variety of candidate positions for each dataset. When using IDA to detect a putative indel in a new dataset the question of the choice of a threshold *f_THRESH_* for the detector function arises. For step 1 of IDA, *f_THRESH_* is set considering the desired computation time and sensitivity of step 2. Unlike classical statistical decision problems, our algorithm is very tolerant of false positives from the first step of the algorithm because false positives are eliminated by SW in the second step. Thus, traditional considerations such as false discovery rate or family-wise error rate are not optimal approaches in determining an optimal *f_THRESH_*. Instead, we suggest the following approach. First, take the top *N* (e.g. *N*=*200*) positions with highest value of *f*(*k*) and pass these through the second step. If at least one of the positions is confirmed, take the next set of *N* positions and repeat. This process is iterated until indels are no longer confirmed.

### Computation complexity and tradeoff

Step 1 of our algorithm is rapid and requires *O*(*T*) time with *T* being the length of the reference sequence. The computational time cost of the step 2 depends on the number of candidate indel locations generated by the first step. By having a sufficiently large threshold value, *α*, we can reduce the false discovery rate (FDR) and therefore, reduce the number of candidate locations. Letting *γ* denote the fraction of reference sequences identified, *γT* would then be the total number of sites that we would have to match to the non-aligned sequences using SW algorithm. Let there be *R* non-aligned sequences, then the computational complexity of the second pass is *γTRn*, where *n* is the length of a given read. Hence, it is clear that the proposed algorithm will only be fast if *γ* is small. As the frequency of indel occurrences is rare in a typical human genome sequence, *γ* is dominated by the FDR. While we can decrease the FDR by increasing *α*, this may also result in missing a true indel.

### Using the normalized depth as an indicator of homozygous, heterozygous or variable ploidy indels

**Figure 3:**
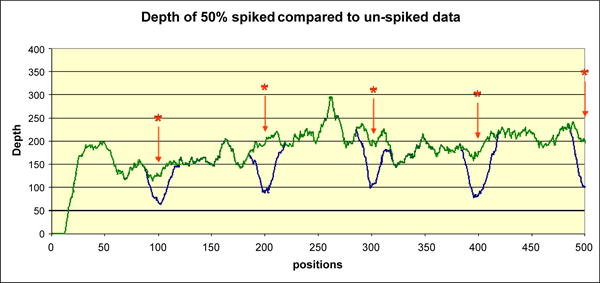
Sequencing depth of phiX174 sequence A portion of phiX174 genome is shown covering positions 1-500 as seen on the Y axis. The X axis represents sequencing depth. The blue curve shows data for the 50% spike ratio, while the green curve shows the depth of the data set without any indels. The positions of the spiked indels, occurring in multiples of 100, are marked by a star (*). The sequencing depth at the indel location dips accordingly.

**Figure 4:**
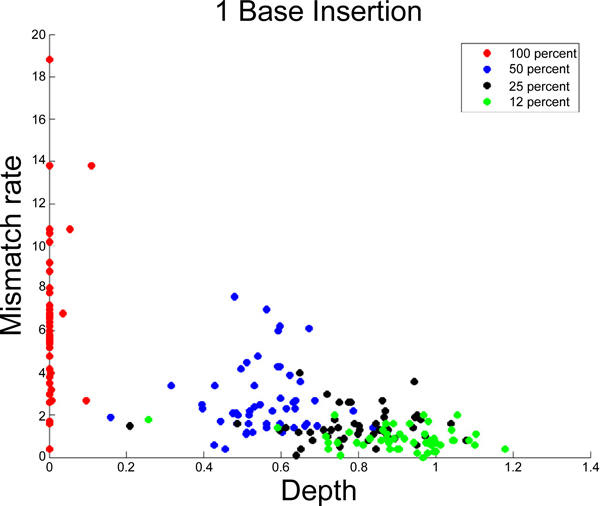
Normalized depth versus mismatch rate for spiked indels Each data point represents a 1 bp insertion. The X axis represents the normalized mismatch rate [1] *m*% and the Y axis represents the normalized sequencing depth *d*%. Results for insertions and deletions 1, 2 and 3 bases in length are similar. Data points are colored accordingly by spike ratio.

We examined the behavior of the normalized depth *d̃* (*k*), and the normalized mismatch rate *m̃*_[1]_ (*k*) in the presence versus the absence of spiked insertions (Figures 3 and Figure 4). As shown in Figure 3, the normalized depth decreases and the normalized mismatch rate increases with the spike fraction. We used these two quantities to build a classifier for distinguishing between homozygous and heterozygous germline indels and estimated the percentage of cells that carry the indel in a data set that simulates a heterogeneous mixed tumor/normal sample. We relied on the same phiX174 data set as previously described.

**Figure 5:**
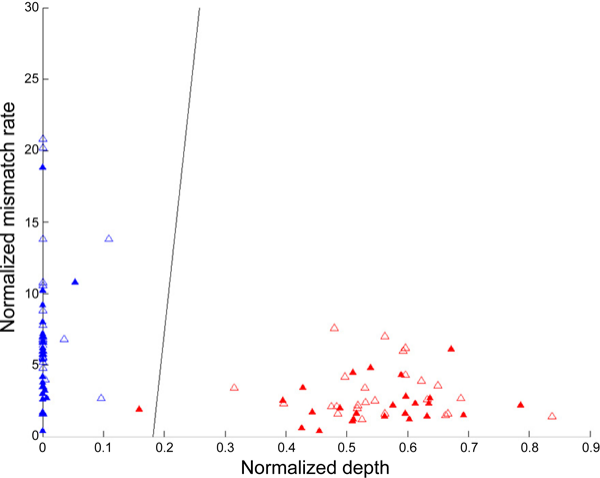
Normalized depth versus mismatch rate for 50% and 100% indels The empty triangles are the training data, the filled triangles are the test data. The X axis represents the normalized mismatch rate [1] *m*% and the Y axis represents the normalized sequencing depth *d*%. The red points are the 50% spiked indels, the blue points are the 100% spiked indels. One of the 50% spiked insertions is misclassified. The black line is the linear classification boundary as defined by the following: 197* [1] *m*% +0.5* *d*% + 77=0.

Consider the case where the sequenced sample is assumed homogeneous and we are interested in classifying a detected indel as homozygous or heterozygous. Given a training phiX174 data set obtained from a 100% and 50% spiked data set as previously described, we fit a linear discriminant classifier that decides whether an indel is 50% or 100% according to its *d̃* (*k*) and *m̃*_[1]_ (*k*) values [4]. The performance of the linear classifier is evaluated by using 50% of the introduced indel locations as training data, and the other 50% as test data. Figure 5 is a scatterplot of the normalized depth versus normalized mismatch rate for both the training and test data. In total there are 50 training and 50 test data points. In both training and test data sets there are 25 50% spiked indels and 25 100% spiked indels. The linear discriminant fitted on the training data was able to classify all but 1 of the 50 test data points correctly. There is one misclassified data point in Figure 5. The reason for the misclassification is that the sequencing depth, in spite of the normalization described in the Methods, was lower than expected. The average misclassification rate based on 100 random 50/50 splits is 0.5 %.

If the sequence data set represents a mixture of normal cells and tumor cells, a quantity of interest would be the proportion *p* of tumor cells that carry an indel. Using the read indel fraction as an indication of *p*, the proportion of an indel (which can be treated as a continuous parameter) can be practically estimated. For this task we propose a logistic regression approach. Let *E*(*k*) be the event that there is an indel at location *k*. We assume the following,

$$\log\text{it}[P(E(k)|\hat{d}(k),{\hat{m}}_{\left[1\right]}(k))]=\alpha +\beta \hat{d}(k)+\delta {\hat{m}}_{\left[1\right]}(k)$$

where logit(*p*)=log[*p* / (1 − *p*)] is the logit function. Given training data obtained from a spike-in study with a continuous range of spike fraction, the parameters *α*, *β*, and *γ* can be estimated. Let *α̂*, *β̂*, and *δ̂* be the estimated parameters. Ten, given a new sample processed through the same experimental protocol, the estimated fraction of the cells containing an indel at location *k* would be denoted by

$$\hat{p}=\exp(\hat{\mu })/[1+\exp(\hat{\mu }=\hat{\alpha }+\hat{\beta }\hat{d}(k)+\hat{\delta }{\hat{m}}_{\left[1\right]}(k)$$

**Figure 6:**
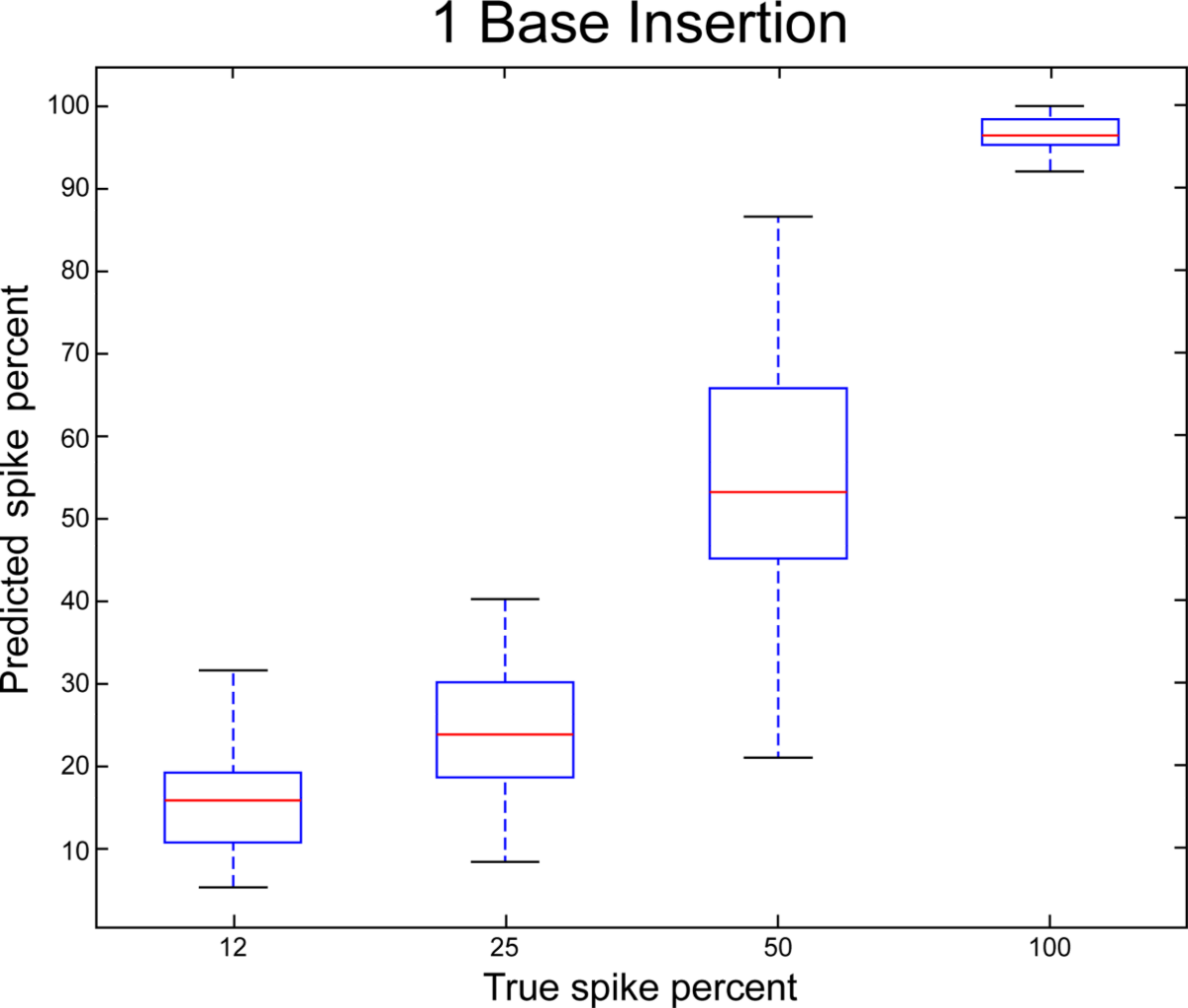
True versus predicted spike fraction on the test data set created with a 1 bp insertion This plot is typical of short insertions of other lengths. The X axis represents the predicted spike faction ˆ*p* and the Y axis represents the actual spike fraction (12, 25, 50, or 100 percent). The average absolute error is 6.9 %, with the largest errors in the 50% and 25 % group.

As illustrated in Figure 5, there is a larger spread in normalized mismatch rate for the lower than for the higher spike fractions. Thus, we expect the estimated fractions *p̂* to be more accurate for the higher spike fractions. As in the case of the linear classifier for homozygous versus heterozygous indels, we divided the spiked data set into training and test, each containing of 50% of the points, and used the model fitted on the training set to estimate the spike fractions for the test set. Figure 6 plots the true fractions versus the estimated values. While the true fractions fall into discrete groups of 12%, 25%, 50% and 100 %, the estimated fractions are continuous. This is a consequence of our model and is desirable in real life settings, for example where a specific sample may contain mixed normal and tumor tissue with arbritrary fractions. The average absolute error in prediction is 6.9 %, with the largest errors in the 50% and 25 % group. The average absolute error in prediction of each class is 6.3%, 8.1%, 12.2% and 4.0 % respectively for the 12%, 25%, 50% and 100% spiked fraction classes.

### Detecting known indels in multiplexed sequencing data of colorectal cancer cell lines containing *TP53* mutations

**Figure 7:**
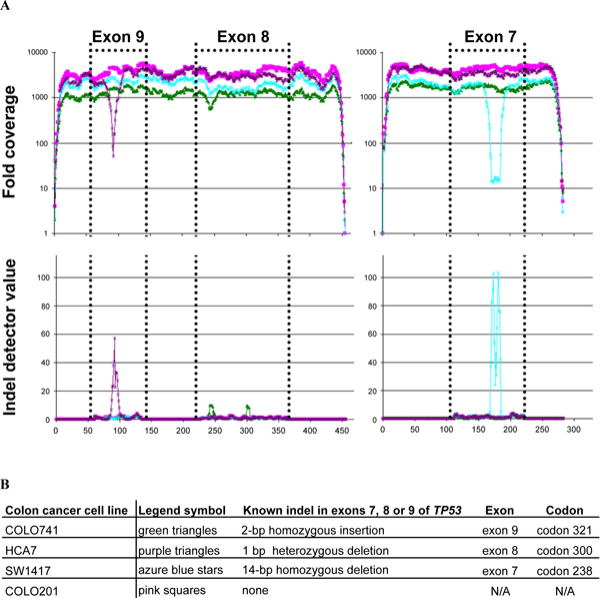
Detection of known indels in four cancer cell lines over exons 7, 8 and 9 of *TP53* Three exons of the *TP53* gene were amplified with two separate amplicons and sequenced. The pink solid box/line indicates COLO201, the green solid triangle/line indicates cell line HCA7, azure blue star/line indicates SW1417 and the purple triangle/line indicates COLO741. A. On the Y axis for the top panel is an indication of the overall depth for each bp position of the amplicons. The exon sequence is delineated by dotted lines. Note that the exon order is based on the human genome build 36.3 and runs in reverse order to the genomic sequence. The Y axis on the bottom panel indicates the indel detector function value. The X axis indicates the bp for each coordinate of the two separate amplicons. The positions of the known indels are distinguished by rises in the indel detector function. B. The presence of the three known indels is detected as an insertion or a deletion of the correct sizes and in the correct positions.

To determine the sensitivity of IDA for indel mutations from human genomes, we sequenced *TP53* exons 7, 8 and 9 from three colorectal cancer cell lines containing known indels in these exons [5]. A cancer cell line not containing any indels in these exons was also sequenced as a control. These exons were PCR amplified, pooled for each sample and subsequently sequenced in an Illumina Genome analyzer. The sequence reads were aligned and submitted to IDA (Figure 7). The algorithm correctly predicts deletions of 1 and 14 bases in lines HCA7 and SW1417 respectively. It also predicts a 2-base insertion in Colo741. Indels present in SW1417 and Colo741 are known to be heterozygous and cause a very deep drop in the sequencing depth. The one base deletion present in HCA7 is heterozygous and causes a much shallower drop in the fold coverage. The detector function however does rise above background even in HCA7 and the presence of the deletion is confirmed by SW. We also tested an alternate confirmation method. We simply identified the sequence reads perfectly matching a 6 base string upstream and downstream of the candidate positions and checked whether those two strings are equidistant in the reference and in the majority of the sequence reads derived from that region. The difference between these distances is the size of the indel. This method is faster and more direct than SW and is preferable when analyzing large datasets.

## Discussion

For next generation sequencing, detecting indel variants from targeted resequencing data has challenges regarding accurate detection. In the case of complex samples where an indel is represented at lower fractions to compared to the wild type reads, sensitive and accurate detection of indels is difficult. Some alignment programs are optimized for fast alignments and only detect mismatches. Others can directly detect indels in addition to mismatches but do so at a cost of a very large increase in time and memory requirements. We have described here a novel algorithm, IDA, which uses two specific features produced by rapid alignments programs to vastly reduce the computational requirements and identify with high sensitivity and specificity of variable sizes including indels less than 20 bp in size. Using single read sequences, the algorithm uses the result of an initial mismatch-only alignment as input. Given its flexibility it can be readily incorporated in a single read sequence analysis pipeline that generates an initial alignment and in the future, we will develop a standalone application for this purpose. While we initially tested our algorithm for detecting indels on sequence reads generated from an Illumina Genome Analyzer, the same approach should be applicable to all other next generation sequencers with short reads.

In terms of computation requirements, IDA was developed and run locally on a standard PC (Windows XP, 2.1GHz, 1 GB RAM). The first step of the algorithm runs in linear time proportionate to reference sequence length, and runs almost instantaneously on the phiX174 genome. In processing a full size data set representing a sequencing lane from an Illumina instrument (e.g. 13 million reads), the second step of the algorithm takes approximately 50 seconds per candidate position tested. It detects both deletions ranging in size from 1 bp to the size of the read length and insertions up to 3 bp in length. Using a straightforward linear classifier, we show that it is possible to discriminate between heterozygote versus homozygote indels. There are obvious advantages to detecting heterozygous indels based on recent surveys of the diploid human genome among other organisms with more complex genomes [6]. We feel that this basic level of genotype discrimination represents a potentially useful and important application of our algorithm. We also demonstrate that IDA can detect indels present in only a portion of the sample, suggesting it will be possible to detect indels in mixed samples. This represents a major advantage of IDA for analyzing sequencing data sets derived from heterogeneous sources such as tumors where a fraction of normal tissue among the tumor creates complex fractional representations of indels.

A major limitation of IDA is that insertions approaching the read length in size are detected by the first step of the algorithm but are not confirmed by SW. To improve this deficiency, one could conduct a localized assembly, for which there are a variety of tools available for reads [1]. For future development of IDA, we plan on testing localized assembly using some of the current tools such as VELVET [7].

## Supplementary Material

Supplement

## Figures and Tables

**Table 1:** Results of 1 bp insertion spike-in study on phix174 sequence reads A subset of sequence reads representing approximately 76,000 sequence reads were obtained from one lane of sequencing of phiX174. Insertions and deletions were introduced in 100%, 50% and 25% of the Sequence reads covering discrete locations in the phiX174 genome. These indels were introduced at positions 100 bp apart starting at position 100 and ending at position 5000 of the phix174 genome. As noted from columns left to right, for insertions and deletions we report separately the length of the introduced mutation, the percentage of sequence reads with an introduced mutation, the number of tested candidates, the number of true positive (TP) indels among the tested candidates, the number of predictions made by the second step of the algorithm, the sensitivity and the specificity of the prediction.

Insertions							Deletions						
mutation size	spike ratio	candidates	TP in candidates	number of predictions	sensitivity	specificity	mutation size	spike ratio	candidates	TP in candidates	number of predictions	sensitivity	specificity
	
1	100	100	28	28	0.56	1	1	100	100	30	33	0.6	0.9996
		200	45	45	0.9	1			200	44	47	0.88	0.9996
		400	50	51	1	1			400	50	58	1	0.999
	
	50	100	18	17	0.32	1		50	100	17	19	0.3	0.9994
		200	36	35	0.68	1			200	36	40	0.72	0.9994
		400	46	48	0.92	1			400	48	54	0.96	0.999
	
	25	100	7	5	0.08	1		25	100	7	8	0.08	0.9992
		200	21	19	0.36	1			200	19	18	0.28	0.9992
		400	36	33	0.64	1			400	41	41	0.72	0.999
	
2	100	100	25	27	0.5	1	2	100	100	25	29	0.48	1
		200	43	48	0.86	1			200	42	52	0.84	1
		400	50	69	1	1			400	49	83	0.98	0.9996
	
	50	100	18	17	0.32	1		50	100	15	22	0.28	0.9996
		200	31	34	0.6	1			200	31	44	0.62	0.9994
		400	46	52	0.92	1			400	45	65	0.88	0.9994
	
	25	100	5	3	0.04	1		25	100	5	9	0.04	0.9994
		200	19	15	0.28	1			200	18	22	0.28	0.9994
		400	31	33	0.58	1			400	29	37	0.54	0.9992
	
3	100	100	29	30	0.56	1	3	100	100	28	29	0.54	1
		200	41	49	0.8	1			200	43	54	0.86	1
		400	50	75	1	1			400	49	81	0.98	0.9996
	
	50	100	21	21	0.36	1		50	100	19	20	0.32	0.9996
		200	34	37	0.62	1			200	34	43	0.66	0.9996
		400	43	55	0.8	1			400	47	69	0.92	0.9994
	
	25	100	8	7	0.1	1		25	100	7	9	0.08	0.9994
		200	19	19	0.3	1			200	21	22	0.32	0.9994
		400	34	39	0.64	0.9998			400	34	45	0.66	0.9994
	
