# Supplementary material for: Identification of Insertion Deletion Mutations from Deep Targeted Resequencing
Source: J Data Mining Genomics Proteomics. Author manuscript; Available in PMC 2014 Feb 7. (PMC3917607; doi:10.4172/2153-0602.1000132)
Supplement: Supplement [file NIHMS519887-supplement-Supplement.pdf]

Supplemental Material

Section 1: Sequence alignment

IDA requires post-alignment data followed by the IDA Matlab-coded script. Both processes can readily be run on a standard desktop or laptop computer. Using a standard laptop (2.1. Ghz, 1Gb RAM), processing the same full size data set (13 million sequence reads, 2 million of which do not align) takes approximately 50 seconds per candidate position tested.

Section 2: ROC analysis of insertion and deletions ranging from 1 to 28 bases

We repeated the analysis presented in Table 1 in the Results section. This time we used a dataset composed of 20,000 28 bp sequence reads derived from a simulated 10.1 Kbp random sequence. (See the Methods section for additional details.) This sequence represents a control for sequence-specific artifacts observed in phiX174. We were able to introduce twice as many indels at the same 100 bp intervals because the simulated sequence is longer than phiX174. This simulated sequence data set served to give additional verification of IDA as well as providing more robust estimates of sensitivity and specificity. To facilitate the comparison with the phiX174 data (Table 1) in which only 50 indels were introduced, we tested an equal number of candidate positions per spiked indel. This represents a maximum of 800 candidate indels per dataset.

Because these sequence reads are generated *in-silico*, we can introduce mismatches modeling sequencing errors at any rate and with any distribution through a given read. Specifically, results were obtained with a dataset containing one mismatch in each 28 bp sequence, corresponding to a ~3% error rate. This is at least double what we observe in real sequencing runs on an Illumina Genome Analyzer I. The mismatches are introduced in the sequence reads according to the approximate distribution observed in real runs; the rate in the last quarter of the sequences (bp 22 to 28) is double that of the previous quarter (bp 15 to 21) and that rate is double the rate of the first half of the sequences (bp 1 to 14).

We investigated longer insertions and deletions. In particular, we were interested in indels approximately equal in size to the read length. We did not explore indels larger than the read length because paired-end, mare-pair sequencing and array comparative genomic hybridization (CGH) approaches are probably more suited to the detection of larger rearrangements.

The ROC results, as shown in Supplemental Table 2, demonstrate that both insertions and deletions in the 1, 2 and 3 bp size range are detected with approximately equal sensitivity. Deletions 12

and 28 bp in length are detected at 62% and 66% sensitivity respectively. This is not significantly lower than the 81%, 74% and 82% achieved for deletions 1, 2 and 3 bp in length. The ability to detect deletions in the length range greater than 3 bp is entirely dependent on setting the Smith - Waterman (SW) algorithm's *gapextend* parameter to zero. If one uses the default value of 8 for *gapextend*, IDA produces the exact same results as those presented here for short deletions (<= 3 bp) but very low sensitivity (<10%) for 12 and 28 bp deletions.

Supplemental Table 1: Results of indel spike-in study on simulated sequence read. TP, FN, TN, FP are true positives, false negatives, true negatives and false positives respectively.

mutation type	mutation size	candidates	TP in candidates	number of predictions	TP	FN	TN	FP	sensitivity	specificity
insertion	1	100	28	28	28	72	9200	0	0.28	1
		200	51	50	50	50	9200	0	0.5	1
		400	64	61	61	39	9200	0	0.61	1
		800	86	73	73	27	9200	0	0.73	1
	2	100	29	30	28	72	9200	0	0.28	1
		200	49	53	47	53	9200	0	0.47	1
		400	69	75	65	35	9200	0	0.65	1
		800	89	84	72	28	9200	0	0.72	1
	3	100	30	30	28	72	9200	0	0.28	1
		200	49	49	47	53	9200	0	0.47	1
		400	71	66	64	36	9200	0	0.64	1
		800	87	79	73	27	9200	0	0.73	1
	12	100	29	0	0	0	0	0	0	0
		200	48	2	2	98	9200	0	0.02	1
		400	67	2	2	98	9200	0	0.02	1
		800	85	2	2	98	9200	0	0.02	1
	28	100	26	0	0	0	0	0	0	0
		200	45	0	0	0	0	0	0	0
		400	64	1	1	99	9200	0	0.01	1
		800	84	1	1	99	9200	0	0.01	1
deletion	1	100	32	32	32	68	9200	0	0.32	1
		200	49	48	48	52	9200	0	0.48	1
		400	71	69	69	31	9200	0	0.69	1
		800	90	83	83	17	9200	0	0.83	1
	2	100	34	37	33	67	9200	0	0.33	1
		200	49	59	47	53	9200	0	0.47	1
		400	70	85	66	34	9200	0	0.66	1
		800	89	102	77	23	9200	0	0.77	1
	3	100	30	32	30	70	9200	0	0.3	1
		200	54	57	54	46	9200	0	0.54	1
		400	71	82	70	30	9200	0	0.7	1
		800	91	106	83	17	9200	0	0.83	1
	12	100	31	21	18	82	9200	0	0.18	1
		200	51	34	30	70	9200	0	0.3	1
		400	71	54	48	52	9200	0	0.48	1
		800	94	76	67	33	9200	0	0.67	1
	28	100	21	22	20	80	9200	0	0.2	1
		200	33	32	30	70	9200	0	0.3	1
		400	57	55	51	49	9200	0	0.51	1
		800	75	73	67	33	9200	0	0.67	1

Both 12 and 28 bp length insertions are not detected successfully, with a sensitivity of 2% and 1% respectively. It should be noted that the failure to detect insertions greater than or equal to 12 bp is entirely due to a lack of SW confirmation in step 2 of IDA. Step 1 of IDA readily detects indels of all size up to the read length. This can be seen by comparing the values in columns "TP" and "TP in candidates" across the various indel sizes. This is also vividly illustrated by comparing the values achieved by the detector function *f(k)* around insertions of varying sizes (Fig. 2A). We postulate that confirming the 12 and 28 bp insertions will likely benefit from other approaches. The different challenges of confirming longer deletions or longer insertions are illustrated in the schematic below.

Supplemental Figure 1: ***Challenges facing confirmation of long indels using IDA.*** *Top panel illustrates the case of deletions while the bottom panel illustrates the case of insertions. In each case the top line is the reference segment used. The sequence read fragments are shown below the reference. The SW algorithm readily aligns sequence reads containing deletions of any size provided that the reference segment extends past the deletions and that the 'extend gap' penalty is set to zero (top panel). For insertions larger than the read length however, no single sequence read can align at both ends to the reference segment (bottom panel). In that case we propose to assemble the sequence reads in order to bridge the gap caused by the insertion.*

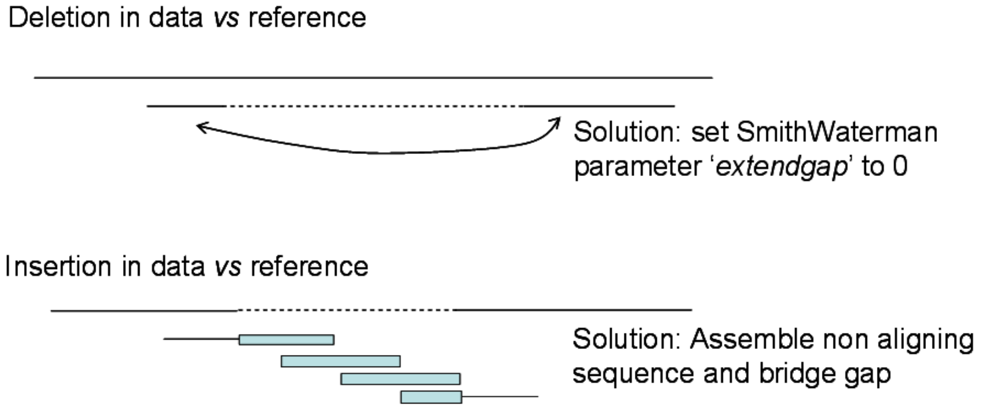

Section 3: How to choose the window size parameters w_d and w_m

We examined the effect of the choice of the parameters w_d and w_m on the specificity and sensitivity of the first step of the indel detection algorithm. We assess the accuracy of step 1 of IDA by determining whether the true spiked positions are contained in the reference sequences generated by the candidates. The reference sequences are windows of size 2*40 surrounding any position *t* for which *f(t)* is above the threshold. Thus, for any given cutoff value f_{THRESH}, we define as a false positive any position *t* that is at least *40* bp away from the spiked positions and that has $f(t) > f_{THRESH}$. We define as false negative any position *t* that is within *40* bp from any spiked position and that has *f(t)* below the threshold. (40 bp is dependent on the read length – longer read lengths would require a value greater than 40.) The sensitivity is the number of positives divided by the total number of positions that are within L bases from the spiked positions. We define false positives/negatives this way because any indel that is within L bases of a position identified in the first step will be aligned in the second step by Smith Waterman.

Thus, for any setting of w_d and w_m, we can compute the ROC curve by varying the value of f_{THRESH}. Here, we examine how the ROC curve depends on the length of the indel and whether the indel is an insertion or deletion. We would like to choose values of w_d and w_m that have an adequate sensitivity and specificity across a wide range of indel lengths.

Figure S3a shows the ROC curve for insertions of length 3 for spike ratio of 25%. As expected by examining Figure 1, the width of the drop in depth does not depend on the length of the insertion, and is expected to span exactly 2 read lengths. Thus, intuitively, the values of w_d and w_m should be at least $2L$, as smaller values would cause part of the signal to be normalized out. The ROC curves confirm this: As w_d and w_m increases from 10 to 60, the ROC curve rises. Then it stabilize and further increases in window size does not seem to have much effect.

Figure S3b shows the ROC curve for deletions of length 3 bp for a spike ratio of 25%. Deletions are more complex than insertions, since longer deletions would require longer window sizes. This is seen by a greater separation between the curves shown in S2b relative to those in S2a. Here, we show that for a deletion of length 3 bp the optimal window size must be a length of $2L$, and that performance does not degrade with further increases to window size.

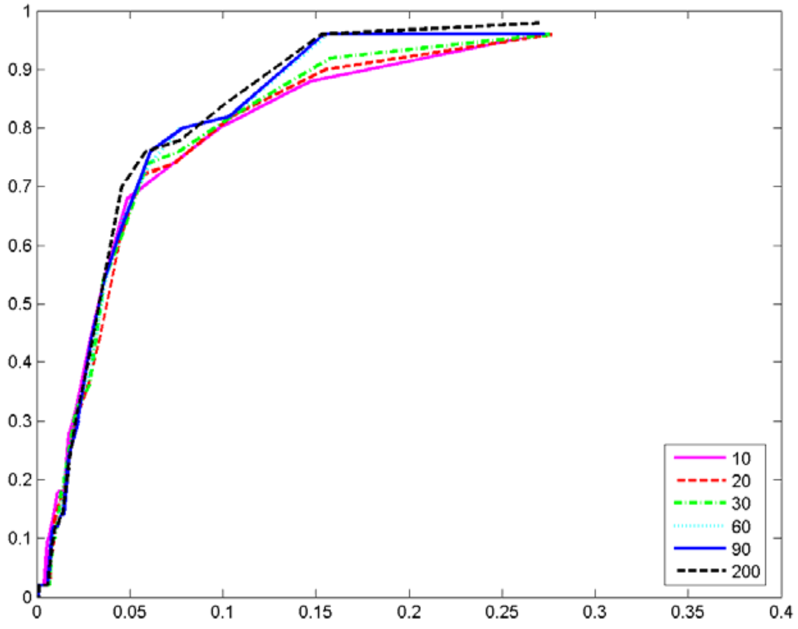

Figure S3a: ROC curve for varying window width for 25% fraction insertions. The legend lists the window widths examined.

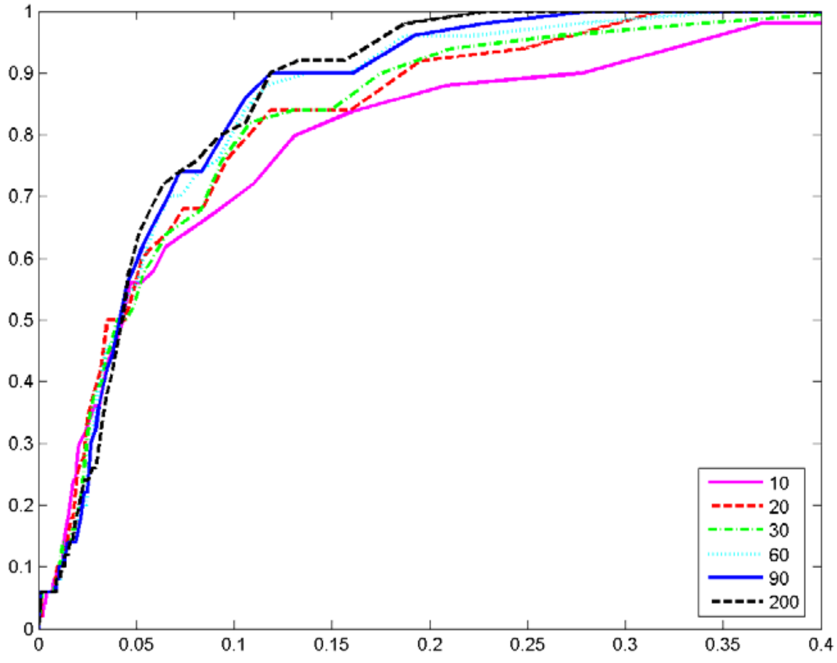

Figure S3b: ROC curve for varying window width for 25% fraction deletions. The legend lists the window widths examined.

Section 3: PCR amplification of exons 7, 8 and 9 of *TP53*

Supplemental Table 2: *TP53* primer sequence information

Primer name	Exon	Primer sequence	PCR product size (bp)	M13 sequence	Complete primer sequence
TP53_07F	7	ctgcttgccacaggtctc	283	TGTAAAACGACGGCCAGT	TGTAAAACGACGGCCAGTctgcttgccacaggtctc
TP53_07R	7	tggatgggtagtagtatggaag	283	CAGGAAACAGCTATGACC	CAGGAAACAGCTATGACCtggatgggtagtagtatggaag
TP53_08-09F	8, 9	gttgggagtagatggagcct	455	TGTAAAACGACGGCCAGT	TGTAAAACGACGGCCAGTgttgggagtagatggagcct
TP53_08-09R	8, 9	ggcattttgagtgttagactg	455	CAGGAAACAGCTATGACC	CAGGAAACAGCTATGACCggcattttgagtgttagactg
